# Tripartite symbiosis of plant-weevil-bacteria is a widespread phenomenon in the Negev Desert

**DOI:** 10.1038/s41598-018-20828-w

**Published:** 2018-02-05

**Authors:** Nitsan Bar-Shmuel, Elena Rogovin, Shimon Rachmilevitch, Ariel-Leib-Leonid Friedman, Oren Shelef, Ishai Hoffmann, Tamir Rosenberg, Adi Behar, Reut Shavit, Fengqun Meng, Michal Segoli

**Affiliations:** 1 Mitrani Department of Desert Ecology, The Swiss Institute for Dryland Environmental and Energy Research, The Jacob Blaustein Institutes for Desert Research, Ben-Gurion University of the Negev, Midreshet Ben-Gurion, Israel; 2 French Associates Institute for Agriculture & Biotechnology of Drylands, The Jacob Blaustein Institutes for Desert Research, Ben-Gurion University of the Negev, Midreshet Ben-Gurion, Israel; 3 The Steinhardt Museum of Natural History, Israel National Center for Biodiversity Studies, Department of Zoology, George S. Wise Faculty of Life Sciences, Tel Aviv University, Tel Aviv, Israel; 4 Kimron Veterinary Institute, Division of Parasitology, Beit-Dagan, 50250 Israel

## Abstract

The weevil *Conorhynchus palumbus* develops in a mud chamber affixed to the roots of the summer annual plant *Salsola inermis* in the Negev Desert of Israel. The weevil carries nitrogen fixing bacteria, and evidence suggests that plants with weevils utilize the fixed nitrogen. To characterize the distribution, abundance and significance of this unique interaction, we surveyed *Salsola* plants in 16 sites throughout the Negev Desert. We excavated ~100 plants from each site, recorded the presence of weevils in their roots, and characterized the soil properties in each site. Weevil mud chambers were present in all of the sampled sites and their abundance was positively correlated with soil nitrogen content and with plant size, and negatively correlated with soil grain-size. Intriguingly, we found two additional weevil species–*Menecleonus virgatus* and *Maximus mimosae*–residing in mud chambers on *Salsola* roots, and found one additional *Salsola* species–*S. incanescens*–accommodating weevils. Nitrogen fixing bacteria were found in weevil larvae of the two additional species and at multiple sites. Overall, our findings suggest that potentially beneficial associations between weevils and plants may be more common than previously acknowledged, and may play an important role in this desert ecosystem.

## Introduction

The geo-chemical properties of desert soils, along with the sporadic rainfall and high temperatures, reduce the availability of water and nutrients for desert organisms^1–3^. These factors affect the distribution, abundance and diversity of flora and fauna in desert ecosystems^4^. Organisms’ survival in these regions involves a constant struggle to obtain limited resources, often requiring specialized morphological, physiological and behavioral strategies^5^.

Symbiotic interactions may offer ways to overcome harsh desert conditions. In particular, mutualistic associations between plants and animals are common, providing their participants with varying services (e.g., nutrition, protection, dispersal and pollination). Some well-known examples include acacia trees, such as the Whistling Thorn (*Vachellia drepanolobium*), that provide ants with nectar secretions and shelter in their bulbous thorns in exchange for defense against large herbivores^6,7^; mistletoes that are aided by birds for direct dispersal of their seeds, while the birds benefit by eating their fruits^8^; and yucca plants that rely on the yucca moth for pollination, while the moth depends on the plant for its development^9^.

Mutualistic interactions with microorganisms are also common in desert environments. These often aid in supplementing limited nutrients for animals living on poor diets, or plants developing on poor soils. For example, some termites that feed on wood or litter rely on the nitrogen fixing bacteria located in their guts for their nutrition^10,11^. In addition, many desert trees benefit from mycorrhizal fungi, which facilitate nutrient and water acquisition through the roots and induce greater tolerance to stress conditions^12^, as well as from nitrogen fixing bacteria, such as rhizobium, that colonize their roots^13,14^.

Despite increasing awareness of the complexity of mutualistic interactions^15,16^, examples of mutualistic relationships that involve plants, animals and microorganisms simultaneously are scarce (but, see ref.^17^). A recently described tripartite underground association between the desert plant *Salsola inermis* (Chenopodiaceae: Caryophyllales), the weevil beetle *Conorhynchus palumbus* (previously known by the junior synonym name *C*. *pistor*, Coleoptera: Curculionidae) and the bacterium *Klebsiella pneumonia* may provide one of the few examples of such mutualism in a desert ecosystem^18^. *S. inermis* is a common annual plant that grows during the summer on poor soils in Israel’s Negev Desert; hence, it is well adapted to both low water and low nitrogen availability^19^. It germinates in the autumn and produces flowers in the summer (June-Sep), when rain water is not available in the Negev^20^. The weevil *C. palumbus* develops within a mud chamber affixed to *S. inermis* roots^18^. The female lays its eggs at the base of *S. inermis* during the spring (Feb–June). The larva constructs a mud chamber attached to the roots during plant growth, and pupates when the plant starts to produce seeds (Aug–Sep). As other food sources are not readily available within the mud chamber, the larva is presumed to gain nutrition from the root, or root exudates, but it does not cause apparent harm to the root or to the plant. In its subsequent imago stage, *C. palumbus* hibernates underground in the winter and emerges in the spring.

Although *S. inermis* plants can grow and reproduce without the weevils, plants with *C. palumbus* in their roots were shown to contain more nitrogen in their tissues and to produce larger seeds^18^, suggesting that the presence of a weevil might provide the plant with nitrogen. In accordance with this hypothesis, the nitrogen fixing bacteria *Klebsiella pneumonia* was identified in the weevil gut, and the activity of the nitrogen fixing enzyme nitrogenase in weevil larvae was demonstrated via chemical tests (acetylene reduction assay)^18^. The combined evidence is highly suggestive that part of the nitrogen fixed inside the weevil is utilized by the plant. Although nitrogen fixing bacteria are known from other insects^21,22^, to our knowledge, this is the only known example of nitrogen fixation in an insect that is potentially beneficial to its host plant.

So far, this association has been described in a single location in the Negev Desert^18^, emphasizing the need for further exploration to determine its distribution, abundance and significance. We took one step in this direction by surveying *S. inermis* in multiple locations throughout the Negev Desert. We tested whether weevil abundance is correlated with environmental factors such as soil properties (e.g., moisture, texture, nutrient levels, salinity, and pH) and plant biomass. In addition, we tested whether additional plant and weevil species may be involved in a similar relationship. *Salsola incanescens* is a related species that often co-occurs with *S. inermis*. Hence, the presence of weevils in this plant’s roots was also examined. Moreover, preliminary surveys suggested that more than one weevil species resides in the roots of *S. inermis*. We identified additional weevils associated with *Salsola* plants and recorded the species composition at the different locations. Finally we confirmed the presence of nitrogen fixing bacteria in the larvae of multiple species and at multiple sites.

Based on the dominance of *S. inermis* in the region and on preliminary observations, we predicted that: (1) this three-way interaction would be widely distributed and involve multiple species; and (2) weevil abundance would correlate with soil properties and plant size.

## Methods

### Study area

**Figure 1:**
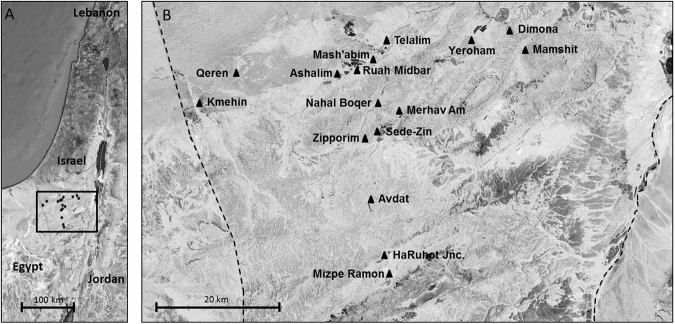
(**A**) Map of study sites (black dots) in the Israeli Negev with a marked section. (**B**) Enlargement of a map section of the Negev Desert Plateau with study sites (black triangles). Exact location of field sites is provided in Appendix A, Table S1. Figure was created using Microsoft PowerPoint 2010 by modifying image from Google Maps (Map data ©2017, Google, Mapa GISrael), retrieved from: https://www.google.co.il/maps/@31.5473592,35.4250579,332960m/data=!3m1!1e3?hl=en.

The focal study area was the Negev Plateau, which is part of the Negev Desert, located at an elevation ranging between 370 m and 520 m above sea level (Fig. 1). The region is characterized by hot, dry summers– with an average maximum daily temperature of ~33 °C in July–August, and cold winters– with an average minimum daily temperature reaching ~5 °C in January–February. Annual precipitation is ~100 mm (data retrieved from the Israel Meteorological Service). Soils in this area are inferior and partly salty.

### Study species

#### The plants

The genus *Salsola* consists of over 100 species found in the dry regions of Asia, Europe, and Africa. In Israel, there are 12 species of *Salsola*, including six annuals. *S. inermis* is a summer annual xerohalophyte confined to somewhat salty loessial soils and to soils disturbed by human activity in Middle Eastern deserts. *Salsola incanescens* is a related species with a similar biology and appearance that often co-occurs with *S. inermis*. An association between *S. incanescens* and root-dwelling weevils has not been described before.

#### The insects

With over 62,000 species, weevils (superfamily Curculionoidea) are the most diverse group of beetles. They are notoriously known as pests because they feed on plants and cause tremendous damage to agricultural crops^23^. The associations between weevils and microorganisms have been studied for more than a decade, and it has been shown that many weevils are dependent on endosymbionts for their nutrition^24,25^. *C. palumbus* belongs to the tribe Cleonini of the subfamily Lixinae, which includes many species feeding primarily or predominantly on roots. Associations between *Salsola* spp. and other weevils of this group have been previously reported; however, these works relate mostly to the harmful effects of the weevils on the plants^26,27^. Evidence suggests that *C. palumbus* provides its host plant with nitrogen, fixed by bacteria residing in the weevil’s guts^18^.

### Field survey

To characterize the distribution and abundance of weevils associated with *S. inermis* and *S. incanescens*, we conducted two field surveys in 16 sites (Fig. 1; Appendix A, Table S1) during 2015. Sampling sites were mainly on loessial or sandy loam soils, found at locations with either a high degree of human disturbance (e.g., road sides) or little to no disturbance (e.g., wadi slopes). *Salsola inermis* occurred in all 16 field sites and in most of them it was more abundant than *Salsola incanescens*, whereas *S. incanescens* was present in seven sites and was more abundant in four of them (Ashalim, Kemehin, Dimona and Mamshit).

The first survey was conducted during August 2015, when the weevils were mostly at their larval stage and hence their species could not yet be morphologically identified. The plants at this time were mostly green and producing flowers, which made it possible to identify the plant species. The second survey was conducted during December 2015, at which time the weevils were mostly at their adult stage and hence could be morphologically identified, and the plants were mostly dry. As our goal was to characterize the plants as well as their associated weevils and to learn about weevil distribution and survival throughout the season, we opted to conduct these two complementary surveys.

In each survey, we excavated around 100 *Salsola* plants per site (*n* = 105 ± 15 plants per sampling), together with their roots, by carefully digging under the plant until the root was fully exposed. Plants were collected along a transect (20 cm wide, length adjusted *in situ*) to minimize sampling biases. Mean (±SD) transect length was 51 m (±38 m), depending on plant density. For each plant, we monitored the presence of weevil mud chambers on their roots and collected the mud chambers. In addition, each plant was assigned to a size category based on canopy diameter (very small: 0–5 cm; small: 5–10 cm; medium: 10–20 cm; large: more than 20 cm). Sampling in the second survey was conducted in a similar manner, several meters (normally less than 5) away from the previous transect.

During the first survey, we also collected three soil samples of about 400 ml from a depth of 10 to 15 cm from each field site for soil analyses. Soil moisture was measured by weighing 100 ml of soil and then drying it (105 °C for 48 hours). Following dehydration, the samples were weighed again and filtered in a 2-mm strainer. Soil water content (WC) was calculated as:

$${\rm{WC}}=1-({\rm{DW}}-{\rm{P}})/({\rm{WW}}-{\rm{P}})$$

where DW = dry soil weight; WW = wet soil weight; and P = weight of particles larger than 2 mm. For total nitrogen and carbon estimations, the samples were dried (60 °C for 48 hours) and filtered in a 2 mm strainer. The percentages of nitrogen and carbon were estimated using an elemental analyzer (FlashEATM1112 CHNS-O Analyser, Thermo Fisher Scientific Inc., UK) with 100 mg of soil and 5 mg of BBOT3 as a standard for calibration. Nitrate and ammonia, organic matter, pH, salinity (Electric Conductivity) and soil texture (% sand, silt and clay) were analyzed according to Sparks *et al*.^28^ at the Gilat service lab, Volcani Center, Israel. With the exception of soil organic matter and pH, soil properties differed significantly among sites (Appendix B, Table S2), suggesting that despite potential variations within each site, our sampling method was effective for characterizing soil properties per site. During the second survey, we also measured the body length and fresh mass of a sample of weevils from each species.

### Presence of nitrogen fixing bacteria

To confirm the presence of nitrogen fixing bacteria in weevils from multiple sites and in the two additional weevil species (*Menecleonus virgatus* and *Maximus mimosae*, see Results section), we collected an additional sample of weevil larvae (*n* = 29) and adults (*n* = 3 of each species) during August-December 2016. Weevils were put in a −80 freezer immediately following collection. DNA was extracted from larvae and adults, using Wizard genomic DNA purification kit (Promega, Madison, WI, USA) according to the manufacturer’s protocols.

Nitrogen-fixing activity is known to occur primarily in the larval stage^18^. However, as larvae cannot be identified morphologically to species, we first used the adult weevils to develop genetic markers for the identification of the weevil species (based on the gene cytochrome c oxidase subunit I [COI). Once the larvae species were identified, DNA of larvae, representing the three weevil species and eleven different field sites (Ashalim, Avdat, Dimona, HaRuhot Jnc., Mash’abim, Merhav Am, Mizpe Ramon, Sede Zin, Tlalim, Yeruham and Zipporim), were sent for bacterial identification using 16S rRNA based pyrosequencing at the Research Resources Center, University of Illinois at Chicago. (See Appendix C, for additional details of the molecular methods, data analysis and sample specifications).

### Statistical analyses

The association between plants and weevils was analyzed both at the site level and at the plant level. At the site level, we correlated weevil abundance (=total number of mud chambers found per site, divided by the total number of plants sampled) with soil parameters (averaged per site). Weevil abundance did not distribute normally; hence, we used the Spearman-Rank correlations. In addition, we compared weevil abundance per site in the first and second survey, using the Wilcoxon matched pairs test. At the plant level, we tested the effect of field site, plant species (in the first survey), and plant size category on the probability that a plant might accommodate a mud chamber, using Generalized Linear Model with a logit link function. Analyses were performed using STATISTICA software.

## Results

### Weevil abundance and distribution

**Figure 2:**
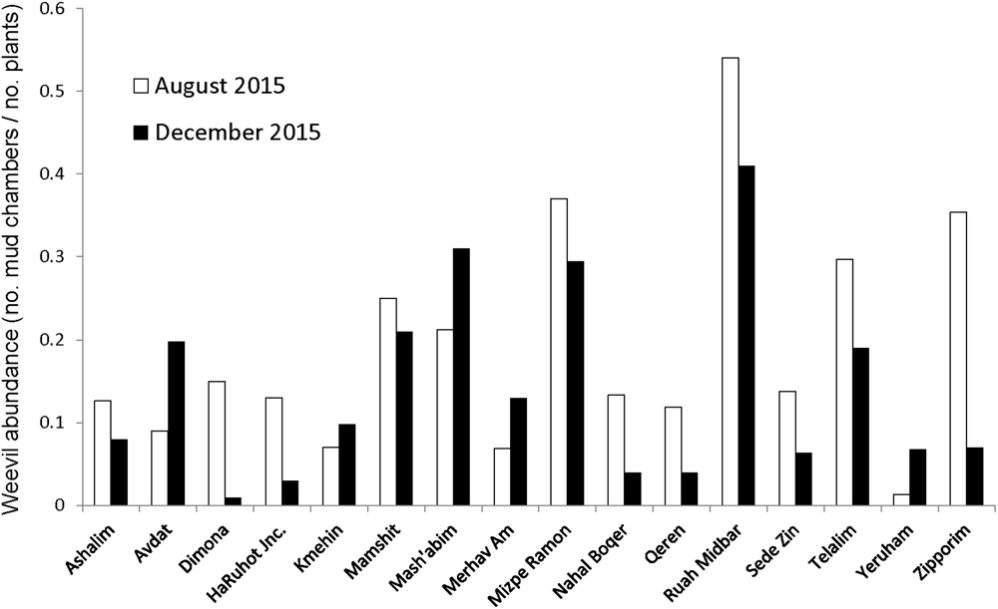
Weevil abundance (=total number of mud chambers found per field site divided by the total number of plants sampled) in the first survey in August (white bars) and the second survey in December (black bars). Weevils were found in all field sites, but their abundance varied (see text for details).

The results of both field surveys (August 2015 and December 2015) indicated that weevils residing on *Salsola* plant roots are widely distributed throughout the Negev Plateau (Fig. 2). In each of the sampled sites, we found at least 2 and up to 54 weevil mud chambers. Weevil abundance (estimated as total number of mud chambers/total no. sampled plants) varied among field sites (Fig. 2), and was higher during the first survey (Wilcoxon’s matched pairs test, *Z* = 3.52, *P* < 0.001), suggesting that some weevils did not survive and in their absence their mud chambers disintegrated throughout the season. In most cases where weevil mud chambers were present, there was a single chamber per plant (*n* = 400), but in some cases, there were two chambers (*n* = 44), three (*n* = 12), four (*n* = 1) and even five chambers (*n* = 3). Most of the mud chambers contained live weevils; in several cases, we found either empty mud chambers (*n* = 21), mud chambers with dead weevils (*n* = 13) or a parasitoid larvae that presumably attacked the weevil (later identified as a bombyliid fly, *n* = 16).

### Correlation with soil properties

**Figure 3:**
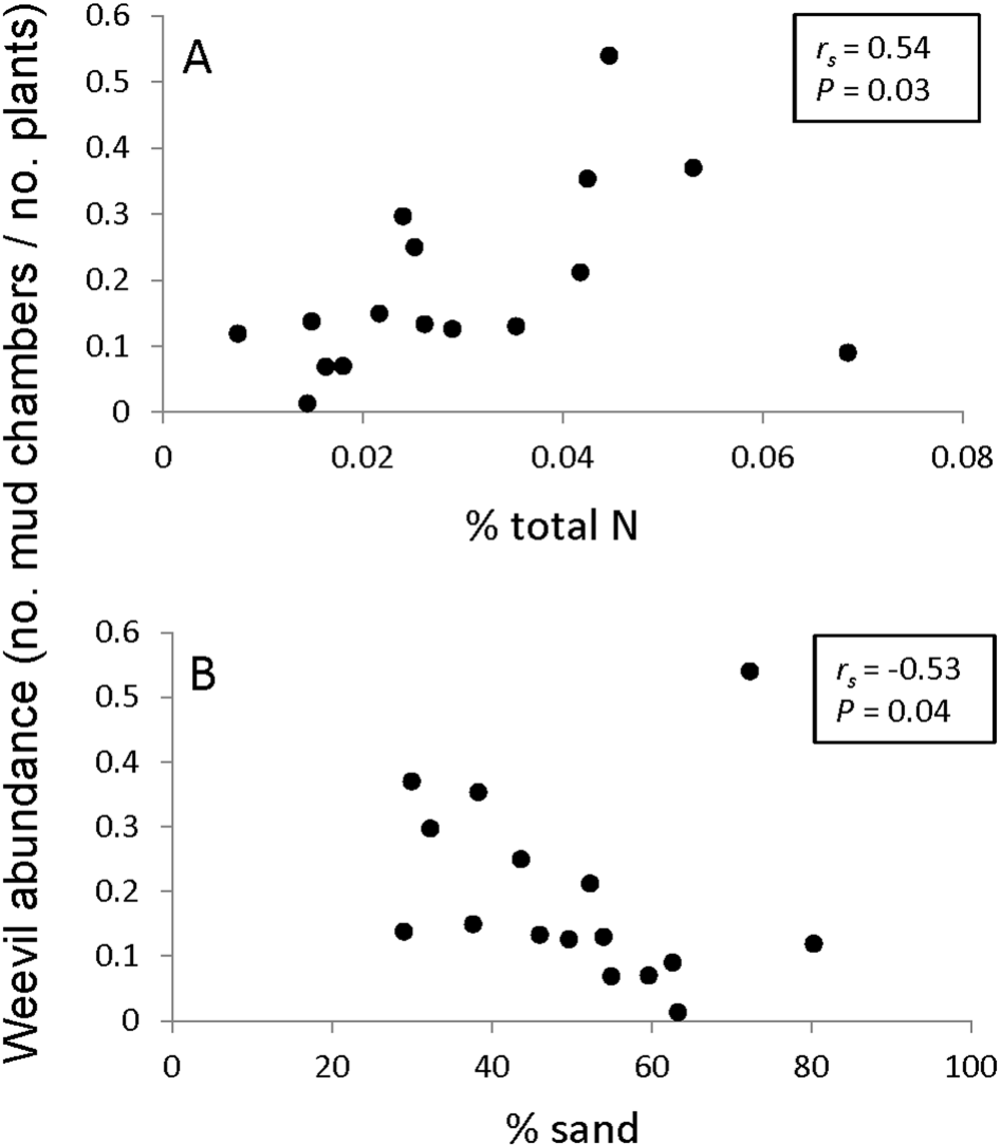
Relationships between soil parameters (averaged per field site) and weevil abundance. Weevil abundance was positively related to soil nitrogen and negatively related to % sand in the soil (see text for details).

Weevil abundance per site (estimated as total number of mud chambers/total number of sampled plants) was significantly and positively related to the percentage of total nitrogen in the soil, averaged per site (Spearman’s rank correlation, *r*_*s*_ = 0.54, *P* = 0.03), and to nitrate levels (Spearman’s rank correlation, *r*_*s*_ = 0.72, *P* < 0.01). Given that nitrate was also directly related to total nitrogen (Spearman’s rank correlation, *r*_*s*_ = 0.68, *P* < 0.01), we only present the correlation of weevil abundance with total nitrogen (Fig. 3A). In addition, weevil abundance was related to soil texture– it was negatively related to the percent of sand in the soil (Spearman’s rank correlation, *r*_*s*_ = −0.53, *P* = 0.036) and positively related to the percentage of silt in the soil (Spearman’s rank correlation, *r*_*s*_ = 0.55, *P* = 0.029), suggesting a higher affinity of weevils to fine-grained soils. Given that the percentage of sand was also directly and negatively related to the percentage of silt, we present only the correlation between weevils and the percentage of sand in the soil (Fig. 3B). Weevil abundance was not related to any of the other measured soil parameters, which were consequently omitted from the graphical representations (Spearman’s rank correlations: soil moisture– *r*_*s*_ = 0.24, *P* = 0.38; carbon percentage– *r*_*s*_ = 0.22, *P* = 0.42; ammonia, organic matter– *r*_*s*_ = 0.16, *P* = 0.55; pH– *r*_*s*_ = −0.32, *P* = 0.23; and salinity– *r*_*s*_ = 0.36, *P* = 0.17).

### Correlation with plant size

**Figure 4:**
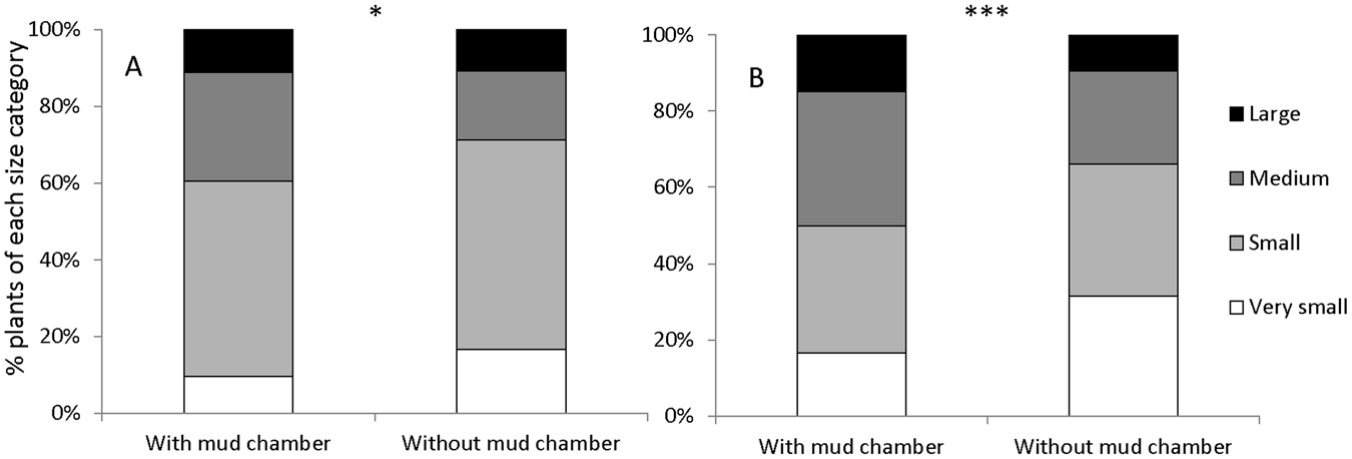
Relationship between presence of mud chamber and plant-size distribution (based on canopy diameter, very small: 0–5 cm; small: 5–10 cm; medium: 10–20 cm; large: more than 20 cm) in the first survey in August (**A**), and the second survey in December (**B**). Plants with a mud chamber were more likely to be of the larger size categories (see text for details).

We found that the probability of a plant having a weevil mud chamber on its roots in the first survey (August 2015) was related to plant size (higher on larger plants than on smaller plants; Generalized Linear Model, *df* = 3, Wald χ^2^ = 10.07, *P* = 0.018, Fig. 4A). The probability was related also to the field site from which the plant was taken (*df* = 15, Wald χ^2^ = 105.37, *P* < 0.001), but not to the plant species (*S. inermis* or *S. incanescens*, *df* = 1, Wald χ^2^ = 1.34, *P* = 0.25). Similarly, in the second survey (December 2015), the probability that a plant would have a weevil mud chamber on its roots was related to plant size (Fig. 4B, *df* = 3, Wald χ2 = 22.66, *P* < 0.001) and to the field site in which the plant was found (*df* = 15, Wald χ2 = 86.71, *P* < 0.001). The probability dependence on plant species was indeterminable for this survey (plant species could not been recognized). The high occurrence of very small plants in the second survey (Fig. 4B compared to 4A) could be explained by the fact that by this time, most plants were already dry.

### Weevil and plant species

**Figure 5:**
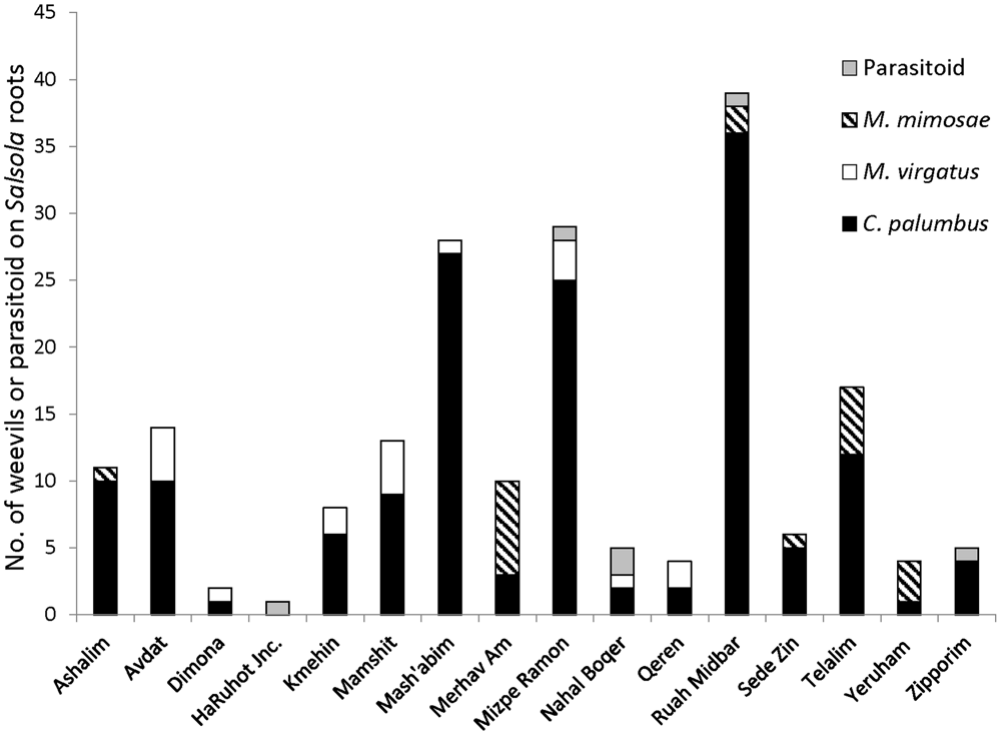
The distribution of the three weevil species and the parasitoid in the different field sites during the second survey (December).

Weevils were found on both *S. inermis* and *S. incanescens*. Plant species had no effect on the probability of a plant having a mud chamber on its roots (see full results in the previous section). Other than the weevil *C. palumbus* (body length: 12.7 ± 1.5 mm [mean ± SD]; wet mass: 0.16 ± 0.05 gr, *N* = 28), which previously has been described in association with *S. inermis*^18^, two additional species of weevils–*Menecleonus virgatus* (body length: 20.3 ± 1.9 mm; wet mass: 0.47 ± 0.15 gr, *N* = 17) and *Maximus mimosae* (body length: 21.2 ± 1.7 mm; wet mass: 0.56 ± 0.13 gr, *N* = 14)– were found in mud chambers affixed to *Salsola* roots. *C. palumbus* was the most common species and was found in all field sites, whereas the two larger species were less common (Fig. 5).

### Presence of nitrogen fixing bacteria in larvae

Based on the genetic markers we identified the majority of the weevil larvae examined as *C. palumbus* (n = 22)– the more common species. The remaining larvae were identified as *M. virgatus* (n = 5) and *M. mimosae* (n = 2). The nitrogen fixing bacteria *Klebssiella spp*. was found in the majority of weevil samples (n = 27), including larvae of the three weevil species and of ten field sites (see Appendix C, Table S5 for details). In addition, we found evidence for the presence of *Enterobacter* and *Citrobacter* (n = 28 and 29 weevils respectively), two additional genera of nitrogen fixing bacteria that are known also from the guts of termites^29^. Examining the relative proportion of nitrogen fixing bacteria inside the weevils revealed that *Citrobacter* was very common (69–99%) while *Klebssiella* and *Enterobacter* were less common (less than 1% of the bacterial community; Appendix C, Table S5).

## Discussion

In this study, we examined the distribution and abundance of weevils that develop in a mud chamber on the roots of *Salsola* plants in the Negev Desert of Israel. *C. palumbus* was previously shown to carry nitrogen fixing bacteria, and evidence suggests that fixed nitrogen is utilized by the host plant, providing a unique example of three-party mutualistic interactions between a plant, an insect and a bacterium. Our findings suggest that the weevils are widely distributed throughout the Negev Desert and that weevil presence is associated with larger plant size. In addition, we found that an additional *Salsola* species (*S. incanescens*) accommodates weevils on its roots, and two additional weevil species (*Menecleonus virgatus* and *Maximus mimosae*) reside in mud chambers in *Salsola* roots. Although these weevils belong to the same tribe (Cleonini) as *C. palumbus*, they are of different genera, suggesting the possibility of convergent evolution. Bacteria identification data demonstrated the occurrence of nitrogen fixing bacteria in weevil larvae of multiple sites and in the two additional weevil species. Our findings support the notion that potentially beneficial associations between weevils and plants are more common than previously acknowledged, and may play an important role in this desert ecosystem.

Despite the occurrence of weevil mud chambers in all of the field sites, weevil abundance was variable. Accordingly, the probability of a plant accommodating a weevil depended on the field site in which it was sampled. These differences may be explained by variations in environmental conditions, such as soil properties, which could affect the weevils directly. Indeed, soil properties are known to affect habitat selection, movement and establishment^30–32^ of insects, and specifically of root herbivores^33^. In addition, soil properties may indirectly affect the interaction between insects and plants^34,35^.

We found that weevil abundance was positively correlated with soil nitrogen, as measured by both total N and nitrate levels. This may suggest that weevil establishment and survival rates are higher in richer soils, potentially because the plants that develop on these soils are more nutritious. Alternatively, higher weevil abundance in a particular site may be the cause rather than the consequence of increased soil nitrogen. This hypothesis may seem a bit far-fetched, however, the high dominance of these annual plants in the field, the large size of the weevils and the extremely low initial nitrogen content in these soils, should be considered. Although biological nitrogen fixation in plant roots (e.g., in legumes) has long been shown to enrich both natural and agricultural soils^36,37^, the relative contribution of nitrogen fixation in insects to soil nitrogen, or the potential use of insects to enrich agricultural soils received little attention. In one example, the presence of ants and termites was shown to increase wheat yield in desert agricultural soils, and this was suggested to be attributed, at least partially, to nitrogen fixation in the termite guts^38^. The possibility that nitrogen fixation in insects enrich desert soils should be further explored.

In addition to soil nitrogen content, weevil abundance was associated with soil texture— its presence was more abundant on silty than on sandy soils. In this case, we find it more probable that weevil establishment and survival was affected by soil texture, rather than vice versa. Soil texture is likely to affect the properties and durability of soil structures built by insects^39,40^. For example, termite mounds are built from soil with a high proportion of fine particles^41^. Similarly, such soils may be preferable for the construction of the weevil mud chamber. Indeed, we observed that mud chambers in sandy soils were more easily broken (though still identifiable) during the excavation process. The weevil larvae are presumed to be mostly immobile; hence, the higher abundance of weevils on silty soils is more likely to be related to habitat selection by the female adult. Similarly, in the Japanese beetle (*Popillia japonica*), females choose a particular soil texture for laying their eggs and avoid laying eggs in sand^42^. Future studies could aim to compare the physical and chemical properties of weevil mud chambers built on different soil types, the habitat selected by adult weevils, as well as larval survival on different soils. We found no association between weevil abundance and other factors, such as soil moisture, total carbon, ammonia, organic matter, pH or salinity. It may be that these variables are less important for the weevils and their interaction with the plants, or that our sample size was not sufficient to detect such associations.

The presence of a weevil in plant roots was associated also with plant size: larger-sized plants were more likely to have a mud chamber, independent of their field of origin. This may be explained by differences in resource availability, root size and architecture among small and large plants, which may potentially affect weevil establishment and survival. Indeed, plant size was shown to affect their interactions with insects in other systems^43,44^. Alternatively, weevil presence could be the cause rather than the consequence of larger plant size. This observation is consistent with the hypothesis that weevil presence contributes to plant growth, perhaps due to their association with nitrogen fixing bacteria.

In conclusion, the interaction between weevils and *Salsola* plants in the Negev Plateau is widely distributed, abundant (at least in some of the sites), and includes multiple species. Further work will aim to expand the sampling to include additional regions within the distribution of this plant in Israel and beyond; test the direct effect of the weevils on plant fitness via manipulative experiments; and determine the relative contributions of nitrogen fixed by bacteria to plant and weevil nutrition, as well as to nutrient cycling in desert soils.

## Electronic supplementary material

Appendices
