# Supplementary material for: Tripartite symbiosis of plant-weevil-bacteria is a widespread phenomenon in the Negev Desert
Source: Sci Rep. 2018 Feb 5;8:2420. doi: 10.1038/s41598-018-20828-w (PMC5799335; doi:10.1038/s41598-018-20828-w)
Supplement: Supplementary file 1 — Appendices [file 41598_2018_20828_MOESM1_ESM.docx]

Tripartite symbiosis of plant-weevil-bacteria is a widespread phenomenon in the Negev Desert

Nitsan Bar-Shmuel^1‡^, Elena Rogovin^1‡^, Shimon Rachmilevitch^2^, Ariel-Leib-Leonid Friedman^3^, Oren Shelef^2^, Ishai Hoffmann^1^, Tamir Rosenberg^1^, Adi Behar^4^, Reut Shavit^1^, Fengqun Meng^1^ and Michal Segoli^1*^

**1** Mitrani Department of Desert Ecology, The Swiss Institute for Dryland Environmental and Energy Research, The Jacob Blaustein Institutes for Desert Research, Ben-Gurion University of the Negev, Midreshet Ben-Gurion, Israel.

**2** French Associates Institute for Agriculture & Biotechnology of Drylands, The Jacob Blaustein Institutes for Desert Research, Ben-Gurion University of the Negev, Midreshet Ben-Gurion, Israel.

**3** The Steinhardt Museum of Natural History, Israel National Center for Biodiversity Studies, Department of Zoology, George S. Wise Faculty of Life Sciences, Tel Aviv University, Tel Aviv, Israel.

**4** Kimron Veterinary Institute, Division of Parasitology, Beit-Dagan 50250, Israel.

‡Both authors contributed equally to this work as the first author.

*msegoli@bgu.ac.il

**Appendix A: Location of field sites**

Table S1. Location of field sites

| Site | Coordinates |
| --- | --- |
| Ashalim | 30°57'51.2"N 34°41'41.5"E |
| Avdat | 30°44'53.6"N 34°46'04.6"E |
| Dimona | 31°02'32.4"N 35°02'42.9"E |
| HaRuchot Jnc. | 30°39'19.5"N 34°47'35.6"E |
| Kmehin | 30°54'48.3"N 34°25'15.0"E |
| Mamshit | 31°00'20.5"N 35°04'28.9"E |
| Mash’abim | 31°01'21.2"N 34°47'50.5"E |
| Merhav Am | 30°53'50.3"N 34°49'32.4"E |
| Mizpe Ramon | 30°37'18.6"N 34°48'15.7"E |
| Nahal Boqer | 30°54'30.6"N 34°46'50.9"E |
| Qeren | 30°57'59.9"N 34°29'27.6"E |
| Ruah-Midbar | 30°58'38.3"N 34°44'15.2"E |
| Sede-Zin | 30°51'50.6"N 34°46'41.2"E |
| Tlalim | 30°59'12.7"N 34°46'06.8"E |
| Yeruham | 31°01'33.1"N 34°58'06.4"E |
| Zipporim | 30°50'55.6"N 34°45'13.2"E |

**Appendix B- Differences in soil properties among field sites**

Table S2. Results of Kruskal-Wallis test comparing soil properties among the different field sites (*N* = 3 soil samples per site).

|  | *df* | Chi square | *P* |
| --- | --- | --- | --- |
| Water content | 15 | 93.22 | < 0.001 |
| % total nitrogen | 15 | 36.73 | 0.001 |
| % total carbon | 15 | 65.80 | < 0.001 |
| Nitrate | 15 | 27.33 | 0.026 |
| Ammonia | 15 | 28.99 | 0.016 |
| Organic matter | 15 | 20.89 | 0.140 |
| pH | 15 | 16.00 | 0.382 |
| Salinity (EC) | 15 | 32.00 | 0.006 |
| % Sand | 15 | 37.33 | 0.001 |
| % Silt | 15 | 37.31 | 0.001 |
| % Clay | 15 | 32.00 | 0.006 |

**Appendix C- Details of molecular methods and results**

DNA Extractions

DNA was extracted using Wizard genomic DNA purification kit (Promega, Madison, WI, USA) according to the manufacturer’s protocols. Prior to the purification, the samples were washed in 3 different solutions: 100% ethanol, 1% dish soap, and phosphate buffer pH 7.0 (Caisson labs, Smith field, IU, USA) in the exact same order and repeated three times. The washed samples were put in a 1.5 ml autoclaved tube and 480 µl 50mM EDTA pH 8.0 (Bio world, Dublin, OH, USA) was added. For cell lysis, samples were homogenized using a pellet pestle and incubated for 2 h at 37 ºC with 120 µl lysozyme (10 mg/ml, Amresco, Solon, OH, USA). The incubation was terminated by 3 cycles of freezing and thawing: freezing in liquid nitrogen for 5 minutes and then thawing at 65 °C for 10 minutes. Samples were then centrifuged at 13,000 g in 4 °C for 10 minutes and the supernatant was removed to allow DNA purification.

Development of genetic markers

The gene cytochrome c oxidase subunit I (COI) was amplified from the DNA of 3 adult weevils from each species (*C. palumbus, M. virgatus* and *M. mimosae*) and sequenced at the Research Resources Center, University of Illinois at Chicago. Sequence outputs were analyzed using clustalW alignment with the Bioedit sequence alignment editor (Tom Hall Ibis Biosciences, Carlsbad, CA, USA) and primers were designed according to the specific sequence of each species, yielding different PCR product sizes. The species identification of larvae was carried out by performing a PCR reaction containing all the marker primers and analyzing the size of the PCR product on a 2% agarose gel. Oligonucleotide primers used in this study were purchased from Sigma-Aldrich and are listed in supplemental Table S3.

PCR was performed using the Bio-ReadyMix (Bio-Lab, Jerusalem, Israel) in a 25-µl reaction volume. The PCR thermal profile consisted of initial denaturation for 5 minutes at 95°C, followed by 35 cycles each of 30 seconds at 95°C, annealing for 40 seconds at X°C, and elongation at 72°C for Y s (X and Y: annealing temperatures and elongation times, respectively, are detailed in supplemental Table S4). A final extension step was performed at 72°C for 10 minutes. Samples of 5 μl from each PCR reaction were separated on a 2% agarose gel (w/v) for 35 minutes at 100 V/cm. Gels were then stained with ethidium-bromide solution (0.5 μg/ml) and photographed with transmitted UV light at 295 nm.

Identification and taxonomic classification of 16S rDNA fragments

Paired-end Illumina reads were merged by using the default settings of FLASH assembler (version 1.2.11; Magoč et al. 2011). The merged sequences were then processed using the default parameters for demultiplexing and quality filtering on QIIME (Quantitative Insights Into Microbial Ecology; version 1.9.0). Quality filtered sequences were clustered stringently using the QIIME UCLUST (Edgar, 2010) module set for a 97% identity threshold (Caporaso et al. 2010). Representative sequences were then chosen for each OUT by choosing the most abundant sequence from the original sequence collection. These default parameters are currently proposed as a standard protocol for 16S taxonomic assignments. Representative OUTs were then aligned against the aligned SILVA rRNA small subunit (SSU) database (release 108, <https://www.mothur.org/wiki/Silva_reference_files> ) with a threshold confidence level of 50% and taxonomic classification was carried out using MOTHER (version 1.36.1) using Wang approach (2007).

Weevil species and the presence of nitrogen fixing bacteria in each sample are presented in table S5.

Table S3. List of primers used in this study.

| **Number** | **Primer name^1^** | **Sequence** |
| --- | --- | --- |
| 1 | LCO1490F | CHACWAAYCATAAAGATATYGG |
| 2 | HCO2198R | AWACTTCVGGRTGVCCAAARAATCA |
| 3 | P 354 F | TTAGTCCCTCTCATACTAGGAGCC |
| 4 | P 354 R | GAAGAGAAAGAAGGAGTAAAATAGCGG |
| 5 | V 235 F | ACTTCCGCCATCTTTAACCTTGT |
| 6 | V 235 R | GGTAGTTCGGTCAGGTGT |
| 7 | M 90 F | GGGGAATAGACCCCGACCA |
| 8 | M 90 R | CTGGAAGAACTGGGAGAGAC |
| 9 | 515Fa | GTGYCAGCMGCCGCGGTAA |
| 10 | 926R | CCGYCAATTYMTTTRAGTTT |

^1^ Forward and reverse primers, are indicated by the suffixes ‘F’ and ‘R’, respectively.

Table S4. Details of PCR reactions

| **Fragment** | **Used primers^1^** | **Annealing temperature (°C)** | **Elongation time (s)** | **Size (bp) of PCR product** |
| --- | --- | --- | --- | --- |
| COI weevils | 1+2 | 51 | 60 | 1100 |
| COI from *Conorhynchus palumbus* | 3+4 | 55 | 30 | 354 |
| COI from *Menecleonus virgatus* | 5+6 | 55 | 30 | 235 |
| COI from *Maximus mimosa* | 7+8 | 55 | 30 | 90 |
| 16s rRNA | 9+10 | 51 | 30 | 411 |

^1^Primer numbers as in supplementary Table S3.

Table S5. Sample specification and bacteria presence/absence and percentage out of the bacterial community based on 16S pyrosequencing.

| Weevil species | Field site | *Klebsiella* | *Enterobacter* | *Citrobacter* |  |
| --- | --- | --- | --- | --- | --- |
| *Conorhynchus palumbus* | Ashalim | **+** (<1%) | **+** (<1%) | **+** (99%) |  |
| *Conorhynchus palumbus* | Ashalim | **+** (<1%) | **+** (<1%) | **+** (99%) |  |
| *Conorhynchus palumbus* | Ashalim | **+** (<1%) | **+** (<1%) | **+** (99%) |  |
| *Conorhynchus palumbus* | Dimona | **+** (<1%) | **+** (<1%) | **+** (89%) |  |
| *Conorhynchus palumbus* | HaRuhut Jnc. | **+** (<1%) | **+** (<1%) | **+** (99%) |  |
| *Conorhynchus palumbus* | Mash'abim | **+** (<1%) | **+** (<1%) | **+** (99%) |  |
| *Conorhynchus palumbus* | Mash'abim | **+** (<1%) | **+** (<1%) | **+** (99%) |  |
| *Conorhynchus palumbus* | Merhav Am | **+** (<1%) | **+** (<1%) | **+** (96%) |  |
| *Conorhynchus palumbus* | Mizpe Ramon | **+** (<1%) | **+** (<1%) | **+** (99%) |  |
| *Conorhynchus palumbus* | Mizpe Ramon | **+** (<1%) | **+** (<1%) | **+** (96%) |  |
| *Conorhynchus palumbus* | Sede Zin | - | **-** | **+** (99%) |  |
| *Conorhynchus palumbus* | Sede Zin | **+** (<1%) | **+** (<1%) | **+** (99%) |  |
| *Conorhynchus palumbus* | Sede Zin | **+** (<1%) | **+** (<1%) | **+** (99%) |  |
| *Conorhynchus palumbus* | Sede Zin | **+** (<1%) | **+** (<1%) | **+** (99%) |  |
| *Conorhynchus palumbus* | Tlalim | **+** (<1%) | **+** (<1%) | **+** (97%) |  |
| *Conorhynchus palumbus* | Tlalim | **+** (<1%) | **+** (<1%) | **+** (95%) |  |
| *Conorhynchus palumbus* | Tlalim | **+** (<1%) | **+** (<1%) | **+** (96%) |  |
| *Conorhynchus palumbus* | Yeruham | **+** (<1%) | **+** (<1%) | **+** (99%) |  |
| *Conorhynchus palumbus* | Zipporim | **+** (<1%) | **+** (<1%) | **+** (99%) |  |
| *Conorhynchus palumbus* | Zipporim | **+** (<1%) | **+** (<1%) | **+** (99%) |  |
| *Conorhynchus palumbus* | Zipporim | **+** (<1%) | **+** (<1%) | **+** (99%) |  |
| *Conorhynchus palumbus* | Zipporim | **+** (<1%) | **+** (<1%) | **+** (99%) |  |
| *Menecleonus virgatus* | Avdat | **+** (<1%) | **+** (<1%) | **+** (93%) |  |
| *Menecleonus virgatus* | Dimona | **+** (<1%) | **+** (<1%) | **+** (89%) |  |
| *Menecleonus virgatus* | Mizpe Ramon | **+** (<1%) | **+** (<1%) | **+** (69%) |  |
| *Menecleonus virgatus* | Mizpe Ramon | **-** | **+** (<1%) | **+** (72%) |  |
| *Menecleonus virgatus* | Yeruham | **+** (<1%) | **+** (<1%) | **+** (98%) |  |
| *Maximus mimosa* | Tlalim | **+** (<1%) | **+** (<1%) | **+** (99%) |  |
| *Maximus mimosa* | Zipporim | **+** (<1%) | **+** (<1%) | **+** (99%) |  |

**References**

1. Caporaso, J. G. et al. QIIME allows analysis of high-throughput community sequencing data. *Nat. Methods* **7**, 335-336 (2010).
2. Edgar, R. C. Search and clustering orders of magnitude faster than BLAST. *Bioinformatics* **26**, 2460-2461 (2010).
3. Magoč, T. & Salzberg, S. L. FLASH: fast length adjustment of short reads to improve genome assemblies. *Bioinformatics* **27**, 2957-2963 (2011).
4. Wang, Q., Garrity, G. M., Tiedje, J. M. & Cole, J. R. Naïve bayesian classifier for rapid assignment of rRNA sequences into the new bacterial taxonomy. *Appl. Environ. Microb*. **73**, 5261-5267 (2007).
